# Supplementary material for: Flexible reuse of cortico-hippocampal representations during encoding and recall of naturalistic events
Source: Nat Commun. 2023 Mar 8;14:1279. doi: 10.1038/s41467-023-36805-5 (PMC9995562; doi:10.1038/s41467-023-36805-5)
Supplement: Supplementary file 1 — Supplementary Information [file 41467_2023_36805_MOESM1_ESM.pdf]

Supplementary Information: Flexible reuse of cortico-hippocampal representations during encoding and recall of naturalistic events

Supplementary Figures

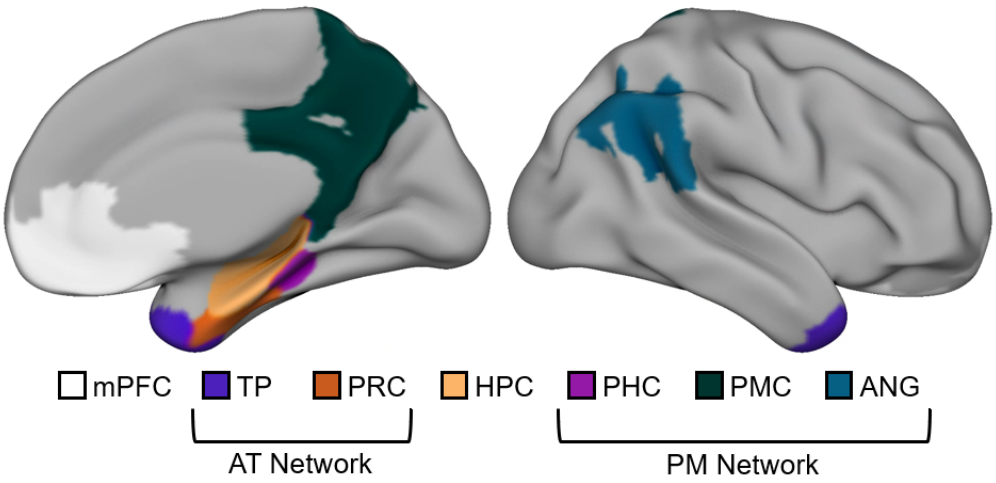

Supplementary Figure 1: Regions of Interest (ROIs). Medial temporal lobe ROIs (HPC, PRC, PHC) were adapted from prior anatomical tracings (Ritchey et al., 2015). Cortical ROIs (mPFC, TP, PMC, ANG) were selected from FreeSurfer segmentations. ROIs are displayed on an inflated brain in MNI space using the Surf Ice software package https://www.nitrc.org/projects/surfice/. COPYRIGHT NOTICE: © Copyright 2007, NITRC. All rights reserved.

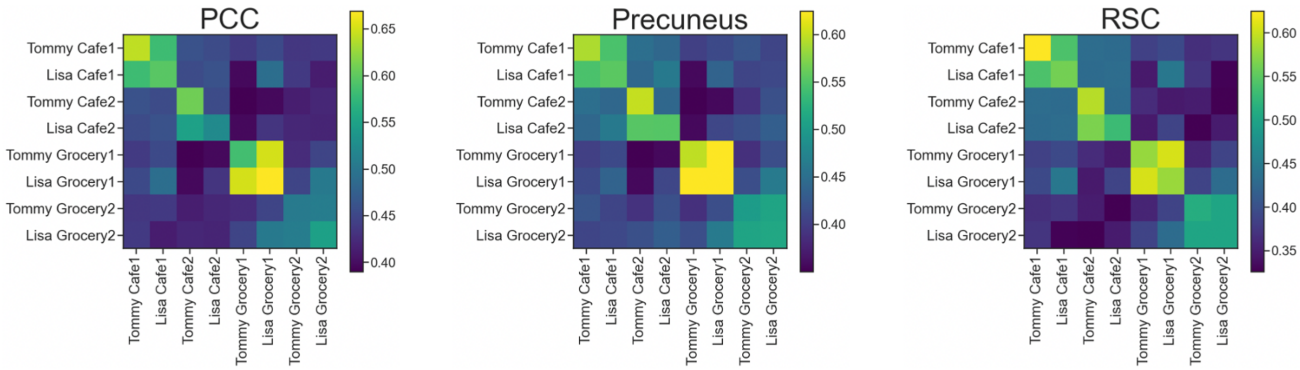

Supplementary Figure 2: ROIs comprising PMC. Event-by-event correlation matrices for individual medial parietal ROIs (posterior cingulate cortex: PCC; precuneus, retrosplenial cortex: RSC) comprising our posterior medial cortex (PMC) ROI, as shown below in Supplementary Figure 3. Correlation matrices are qualitatively very similar to PMC, and correlations between each of these 3 ROIs and our model matrices do not differ statistically from fits observed in PMC (all $p > 0.63$).

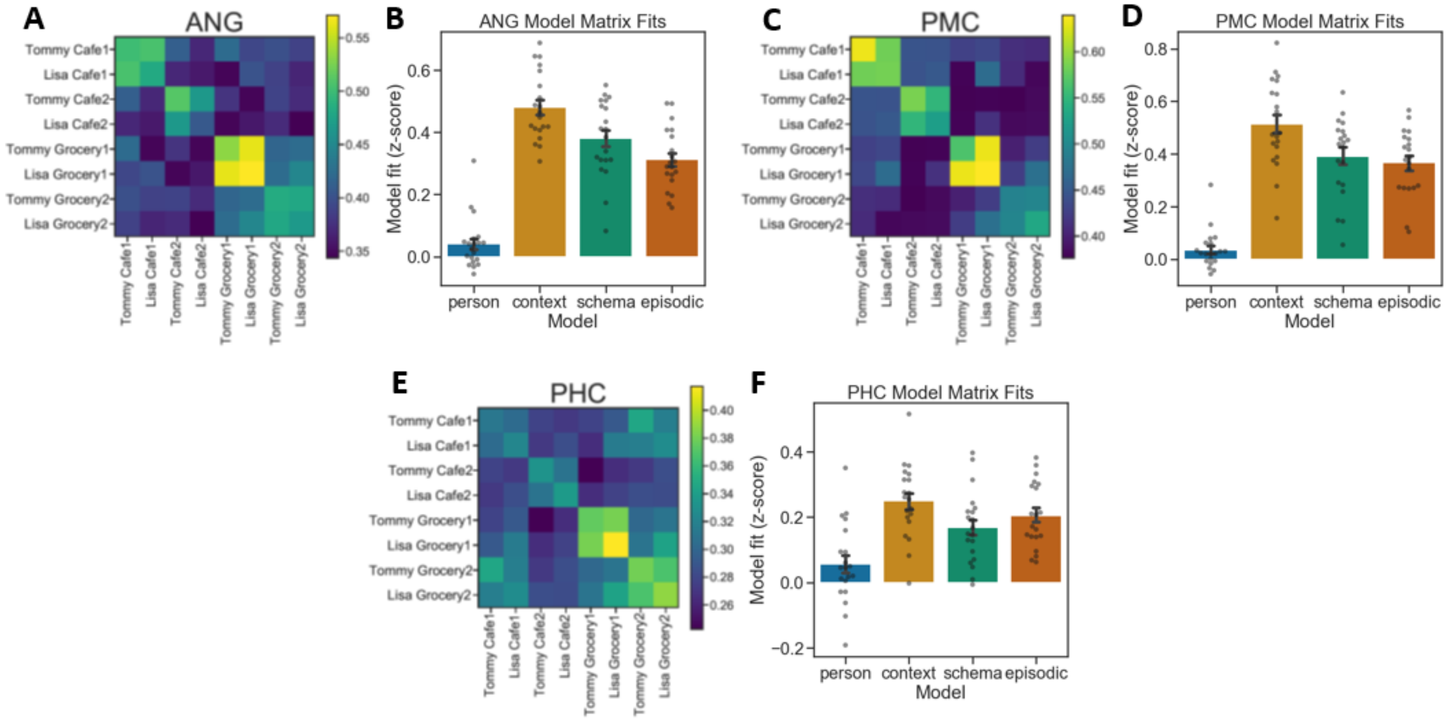

Supplementary Figure 3: Across-event pattern similarity at encoding for individual PM Network ROIs. The strongest fit was observed between the Context matrix and across-event pattern similarity data in (A,B) ANG, (C,D) PMC, and (E,F) PHC.

Supplementary Figure 4: Across-event pattern similarity at encoding for individual AT Network ROIs. The strongest fit was observed between the Person matrix and across-event pattern similarity data in (A,B) PRC and (C,D) TP.

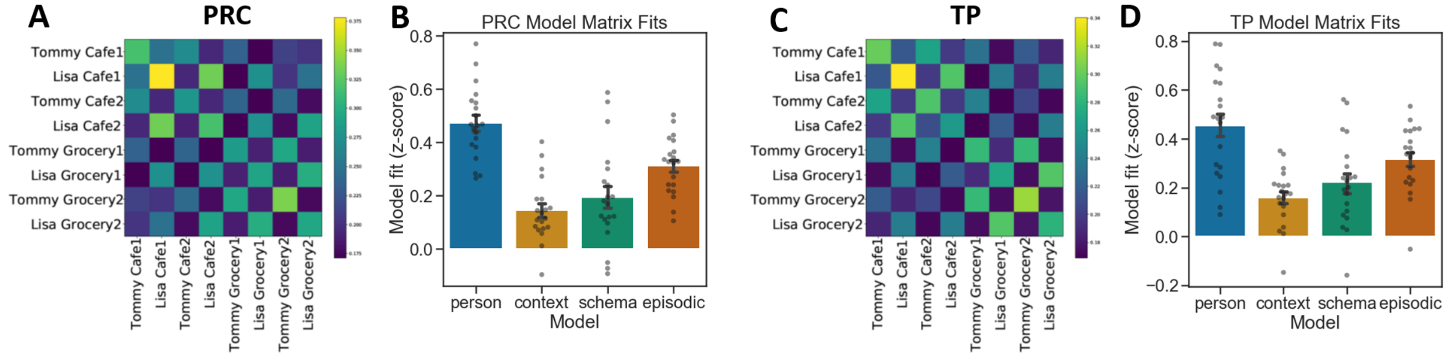

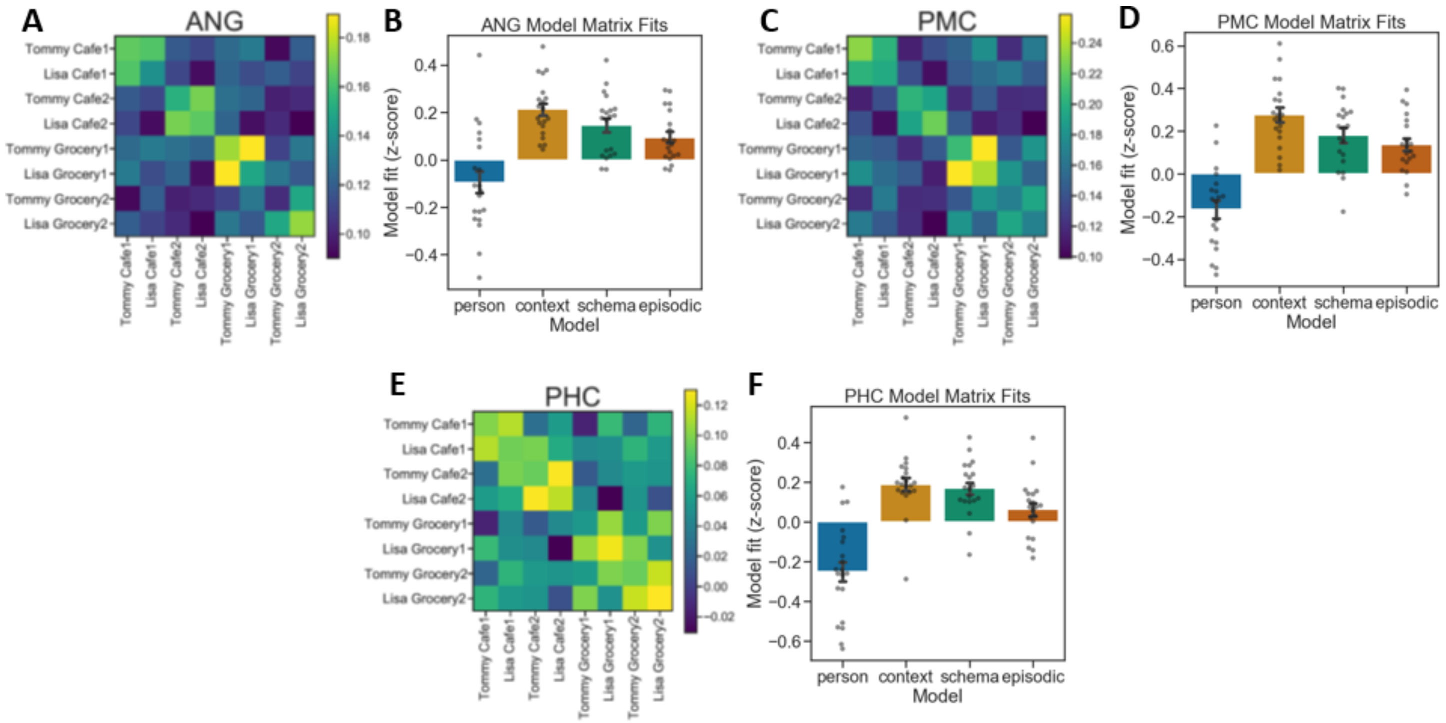

Supplementary Figure 5: Encoding-recall pattern similarity for individual PM Network ROIs. The strongest fit was observed between the Context matrix and across-event pattern similarity data in (A,B) ANG, (C,D) PMC, and (E,F) PHC.

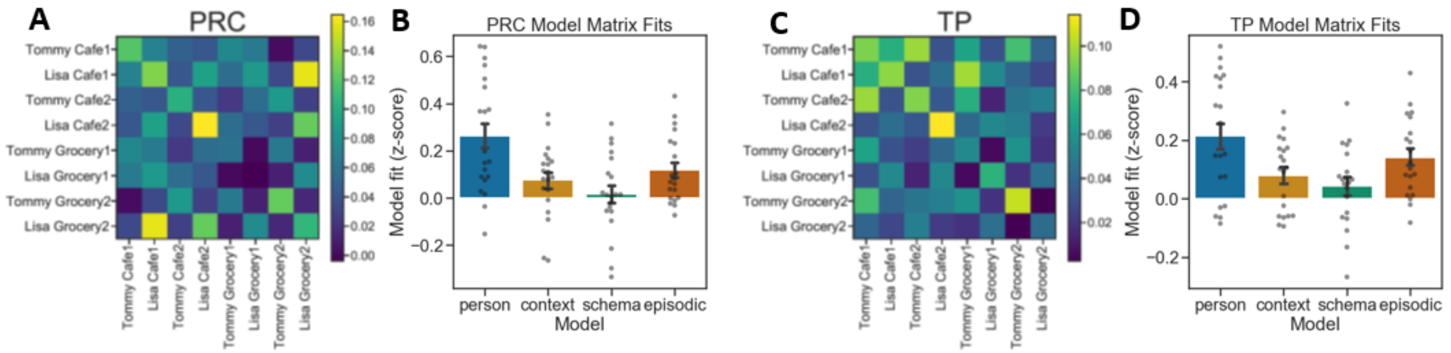

Supplementary Figure 6: Encoding-recall pattern similarity for individual AT Network ROIs. The strongest fit was observed between the Person matrix and across-event pattern similarity data in (A,B) PRC and (C,D) TP.

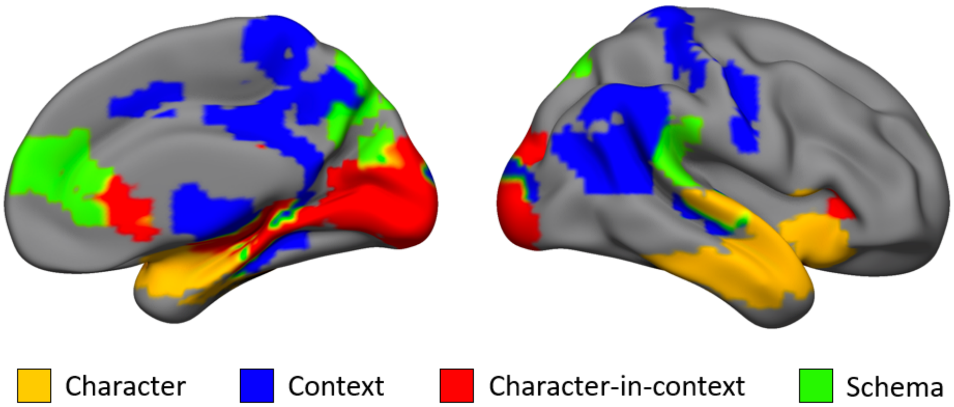

Supplementary Figure 7: Model matrix fits mapped onto cortical surface regions-of-interest. An exploratory analysis was conducted at uncorrected thresholds based on Barnett et al., *PLOS Biology* 2021, which identified 4 cortico-hippocampal networks (PM Network, AT Network, a Medial Prefrontal Network, and a Medial Temporal Network). Model matrix comparisons were conducted in each ROI featured in the Barnett et al., atlas (built on the ROIs featured in Glasser et al., *Nature* 2016). A simple "winner-take-all" analyses was conducted in that, if significant model matrix fits were observed, the strongest-fitting effect was plotted in that ROI. Findings are generally consistent with the more limited ROI-based approach featured in the main analyses. ROIs are displayed on an inflated brain in MNI space using the Surf Ice software package (https://www.nitrc.org/projects/surfice/). COPYRIGHT NOTICE: © Copyright 2007, NITRC. All rights reserved.

Supplementary Note 1

Accurate, detailed, and unbiased memory for events across participants. Memory for videos shown in the scanner was tested in two ways. Participants were scanned during free recall of each film, and following the scan session, they completed a recognition memory test consisting of statements about the events. A standardized protocol was used to score recall data (Cohn-Sheehy et al., 2020) for the number of verifiable details with respect to content in the video clips (see Methods for further details). Participants generally recalled many details about each movie, and each participant recalled information from each event. Across participants, recall scores were significantly above zero (average = 14.81, $t(19) = 11.737$, $p = 1.095e^{-10}$). Recall performance did not differ as a function of context (one-way ANOVA; $F(3,172) = 0.803$, $p = 0.412$) or person (paired t-test; $t(19) = 0.108$, $p = 0.893$) (Table 1, main text). For recognition, we observed significantly greater than chance discrimination of true from false statements about the events (average d' = 1.49, $t(19) = 14.963$, $p = 3.675e^{-12}$), and there were no significant differences as a function of context (one-way ANOVA; $F(3,172) = 0.445$, p = 0 .739) or person (paired t-test; $t(19) = 0.385$, $p = 0.522$). Thus, participants encoded and remembered events in considerable detail, but were not significantly biased to remember or forget details about particular events or particular individuals within those events.

Supplementary Note 2

Breaking PMC into smaller constituent cortical regions. In line with recent work, we included a posterior-medial cortex (PMC) ROI comprising medial parietal subregions. This raises questions of whether constituent cortical regions – namely, retrosplenial cortex (RSC), posterior cingulate cortex (PCC), and precuneus show sufficiently similar correlation profiles to warrant collapsing across these regions. We examined event-by-event correlation matrices for these three constituent ROIs, which were very similar individually to the correlation matrix produced by averaging them together into a PMC ROI (Supplementary Fig. 2). Indeed, contrasting correlation coefficients between each of the three correlation matrices and model matrices against the PMC fits, no correlation approached a significant difference (all $p > 0.63$). This provides support for collapsing these ROIs into PMC, and further supports the notion of medial parietal regions comprising a representationally-coherent PM Network.

Supplementary Note 3

Model matrix results for individual ROIs: encoding. In ANG (Supplementary Fig. 3A), the average pattern similarity correlation matrix across participants was most strongly related to the Context model matrix ($r = 0.794$, $p = 5.193e^{-15}$) with significant correlations also found for the Schema ($r = 0.604$, $p = 1.247e^{-07}$) and Episodic ($r = 0.529$, $p = 6.881\ e^{-06}$) matrices (Supplementary Fig. 3B). The Person matrix correlation was not significant. There was a significant difference among model fits ($F(3,57) = 83.206$, $p < 0.001$), and the Context matrix was a significantly stronger fit to the data than each of the other three ($p_{Tukey} < 0.05$ corrected). Similarly, in PMC (Supplementary Fig. 3C), pattern similarity data most strongly correlated with the Context matrix ($r = 0.856$, $p = 0.586e^{-19}$) and significant relationships were also observed compared to the Schema ($r = 0.657$, $p = 3.679e^{-09}$) and Episodic ($r = 0.607$, $p = 1.048e^{-07}$) matrices (Supplementary Fig. 3D). The Person matrix correlation was not significant. Model fits significantly differed from one another ($F(3,57) = 71.763$, $p < 0.001$), with a stronger Context matrix fit than all others ($p_{Tukey} < 0.05$ corrected). PHC (Supplementary Fig. 3E) also showed this profile, with significant fits to the Context ($r = 0.626$, $p = 3.235e^{-08}$), Schema ($r = 0.413$, $p = 0.001$), and Episodic ($r = 0.521$, $p = 1.019e^{-05}$) model matrices, and a poor fit to the Person matrix ($r = 0.141$, $p = 0.266$) (Supplementary Fig. 3F). There was a significant difference among model fits ($F(3,57) = 16.187$, $p < 0.001$), but only the Context vs. Person matrix fits differed significantly ($p_{Tukey} < 0.05$ corrected).

In PRC (Supplementary Fig. 4A), we found the strongest correlation between the event-by-event pattern similarity matrix and the Person model matrix ($r = 0.838$, $p = 6.117e^{-18}$), though all other model matrix fits were significant as well: Context ($r = 0.249$, $p = 0.047$), Schema ($r = 0.322$, $p = 0.009$), and Episodic ($r = 0.551$, $p = 2.424e^{-06}$) (Supplementary Fig. 4B). Model fits differed significantly ($F(3,57) = 33.42$, $p < 0.001$), and the Person matrix was a significantly stronger fit than all other model matrices ($p_{Tukey} < 0.05$ corrected). Similar results were observed in TP (Supplementary Fig. 4C), where the Person matrix fit the observed data the strongest ($r = 0.0.928$, $p = 2.628e^{-17}$), but the other model matrix fits were significant as well: Context ($r = 0.295$, $p = 0.018$), Schema ($r = 0.41$, $p = 0.001$), and Episodic ($r = 0.588$, $p = 3.301e^{-07}$) (Supplementary Fig. 4D). There was a significant difference among model fits ($F(3,57) = 18.9$, $p < 0.001$), and the Person matrix fit the data significantly better than the Person or Schema matrices ($p_{Tukey} < 0.05$ corrected), but did not differ in a direct pairwise contrast with the Episodic matrix.

Supplementary note 4

Model matrix results for individual ROIs: recall. In ANG (Supplementary Fig. 5A), the average pattern similarity correlation matrix across participants was most strongly related to the Context model matrix ($r = 0.619$, $p = 4.769e^{-08}$) with significant correlations also found for the Schema ($r = 0.401$, $p = 0.001$), Episodic ($r = 0.287$, $p = 0.021$) and matrices, and a significant negative fit to the Person matrix ($r = -0.274$, $p = 0.028$) (Supplementary Fig. 5B). There was a significant difference among model fits ($F(3,57) = 20.4752$, $p < 0.001$), with the Context matrix being a stronger fit than Person or Episodic ($p_{Tukey} < 0.05$ corrected). In PMC (Supplementary Fig. 5C), we found the strongest correlation between encoding-recall pattern similarity data and the Context matrix ($r = 0.635$, $p = 1.771e^{-08}$), though significant correlations were also observed with the Schema ($r = 0.415$, $p = 0.001$), and Episodic ($r = 0.288$, $p = 021$) matrices (Supplementary Fig. 5D). The fit with the Person matrix was significant, but negative ($r = -0.359$, $p = 0.003$). Model fits differed significantly ($F(3,57) = 38.612$, $p < 0.001$), driven by a stronger fit between the Context matrix and other model matrices ($p_{Tukey} < 0.05$ corrected). PHC (Supplementary Fig. 5E) showed the largest correlation with the Context matrix ($r = 0.402$, $p = 0.001$), and was also significantly correlated with the Schema matrix ($r = 0.358$, $p = 0.004$) (Supplementary Fig. 5F). Similar to the prior PM Network regions, PHC showed a significant negative fit to the Person matrix at recall ($r = 0.567$, $p = 1.034e^{-06}$). Model fits differed significantly ($F(3,57) =$

55.481, $p < 0.001$), driven by a significantly poorer fit for the Person matrix than others ($p_{Tukey} < 0.05$ corrected).

In PRC (Supplementary Fig. 6A), we observed significant correlations only with the Person (r = 0.323, p = 0.009) and Episodic (r = 0.267, p = 0.033) model matrices (Supplementary Fig. 6B). The Context and Schema matrix fits were not significant. Model fits differed significantly from one another ($F(3,57) = 2.799$, p = 0.048), but pairwise differences were not significant. In TP (Supplementary Fig. 6C), we observed significant correlations with the Person matrix (r = 0.291, p = 0.003) and the Episodic matrix (r = 0.272, p = 0.005) (Supplementary Fig. 6D). The Context and Schema matrix fits were not significant, and model fits did not differ significantly from one another ($F(3,57) = 1.689$, p = 0.179).

Supplementary note 5

Event-type pattern similarity results for individual ROIs: encoding. Individual regional results are in accord with network-level grouping. We found significant effects of context in each PM Network ROI (ANG: p = 0.015; PMC: p = 0.004; PHC: p = 0.049), which was driven by greater pattern similarity when participants viewed events that occurred within the same context compared to similar (pairwise contrasts: $p < 0.05$) or different contexts (pairwise contrasts: $p < 0.05$). Conversely, we did not find an effect of character ($p > 0.05$). Across both PRC and TP, we found significantly greater pattern similarity when participants viewed the same character compared to events depicting a different character (both $p < 0.001$). Conversely, we found no effect of context ($p > 0.05$).

Supplementary note 6

Event-type pattern similarity results for individual ROIs: recall. Individual regional results are in accord with network-level grouping. Per the same analyses over individual PM Network ROIs, we found a significant effect of context in PMC (p = 0.011) and ANG (p = 0.031), and as with encoding, pattern similarity was higher during recall of events occurring in the same context than in a similar or different context (pairwise contrasts: $p < 0.05$). Effects in PHC were not significant for encoding-recall analyses. This effect was not influenced by character identity ($p > 0.05$). Turning to AT Network ROIs, we found reinstatement of character-related patterns in PRC (p = 0.024) during recall, which were again independent of contextual information ($p > 0.05$) (Fig. 6B). Effects in TP were not significant for encoding-recall analyses.
