# Supplementary material for: Flexible reuse of cortico-hippocampal representations during encoding and recall of naturalistic events
Source: Nat Commun. 2023 Mar 8;14:1279. doi: 10.1038/s41467-023-36805-5 (PMC9995562; doi:10.1038/s41467-023-36805-5)
Supplement: Supplementary file 2 — Reporting Summary [file 41467_2023_36805_MOESM2_ESM.pdf]

nature research

Corresponding author(s): Zachariah Reagh

Last updated by author(s): December 12, 2022

Reporting Summary

Nature Research wishes to improve the reproducibility of the work that we publish. This form provides structure for consistency and transparency in reporting. For further information on Nature Research policies, see our Editorial Policies and the Editorial Policy Checklist.

Statistics

For all statistical analyses, confirm that the following items are present in the figure legend, table legend, main text, or Methods section.

n/a	Confirmed	
☐	☒	The exact sample size (*n*) for each experimental group/condition, given as a discrete number and unit of measurement
☐	☒	A statement on whether measurements were taken from distinct samples or whether the same sample was measured repeatedly
☐	☒	The statistical test(s) used AND whether they are one- or two-sided<br>*Only common tests should be described solely by name; describe more complex techniques in the Methods section.*
☐	☒	A description of all covariates tested
☐	☒	A description of any assumptions or corrections, such as tests of normality and adjustment for multiple comparisons
☐	☒	A full description of the statistical parameters including central tendency (e.g. means) or other basic estimates (e.g. regression coefficient) AND variation (e.g. standard deviation) or associated estimates of uncertainty (e.g. confidence intervals)
☐	☒	For null hypothesis testing, the test statistic (e.g. *F*, *t*, *r*) with confidence intervals, effect sizes, degrees of freedom and *P* value noted<br>*Give P values as exact values whenever suitable.*
☒	☐	For Bayesian analysis, information on the choice of priors and Markov chain Monte Carlo settings
☒	☐	For hierarchical and complex designs, identification of the appropriate level for tests and full reporting of outcomes
☐	☒	Estimates of effect sizes (e.g. Cohen's *d*, Pearson's *r*), indicating how they were calculated

Our web collection on statistics for biologists contains articles on many of the points above.

Software and code

Policy information about availability of computer code

Data collection	Stimuli were presented and behavioral responses were collected using PsychoPy (3.1.5). Video stimuli were uploaded using GoPro Quick, and edited using VLC (3.0.8).
Data analysis	Analysis of behavioral and fMRI data used custom Python code using Anaconda distribution 3.4.2 as well as linear modeling using AFNI version 16.2.07. Specific Python packages used include numpy (1.19.2), pandas (1.2.0), statsmodels (1.12.0), and seaborn (0.11.2). Preprocessing of fMRI data used FMRIPREP (RRID:SCR_016216). Please see Methods in the manuscript for version information for all packages in this version of FMRIPREP.

For manuscripts utilizing custom algorithms or software that are central to the research but not yet described in published literature, software must be made available to editors and reviewers. We strongly encourage code deposition in a community repository (e.g. GitHub). See the Nature Research guidelines for submitting code & software for further information.

Data

Policy information about availability of data

All manuscripts must include a data availability statement. This statement should provide the following information, where applicable:
- Accession codes, unique identifiers, or web links for publicly available datasets
- A list of figures that have associated raw data
- A description of any restrictions on data availability

Data and analysis scripts will be made available upon request to the authors.

Field-specific reporting

Please select the one below that is the best fit for your research. If you are not sure, read the appropriate sections before making your selection.

- [ ] Life sciences
- [x] Behavioural & social sciences
- [ ] Ecological, evolutionary & environmental sciences

For a reference copy of the document with all sections, see nature.com/documents/nr-reporting-summary-flat.pdf

Behavioural & social sciences study design

All studies must disclose on these points even when the disclosure is negative.

Study description	The study involves quantitative experimental approaches. Participants viewed and verbally recalled brief movie clips in the MRI scanner, with the content of movie clips being systematically varied in a factorial design.
Research sample	Participants were recruited from the University of California, Davis and the Davis, California community via flyer and email advertisement. Participants were paid $25 per hour, and gave written informed consent in accordance with the University of California, Davis Institutional Review Board under an approved protocol. Exclusion criteria included magnetic bodily implants, claustrophobia, a history of major psychiatric or neurological disorders, a concussion in the past 6 months, untreated diabetes or hypertension, current drug or alcohol abuse, left-handedness, age outside a range of 18-35 years, and 4 or fewer hours of sleep on the day of data collection. We initially collected 24 participants, and excluded 1 due to falling asleep during MRI scanning, 2 due to excessive motion in the MRI scanner, and 1 due to equipment malfunction during data collection. The final sample of 20 participants had a mean age of 23.4 years (SD = 3.1 years), consisting of 13 self-identified females and 7 self-identified males. This is a fairly representative sample for a cognitive neuroscience study of human memory, with a comparable demographic makeup and sample size to contemporary experiments.
Sampling strategy	Data were sampled randomly on a paid volunteer basis from the University of California, Davis and surrounding community. Sample sizes are sufficient to detect the effects of interests given prior work in this area.
Data collection	Participants arrived at the UC Davis Center for Neuroscience, and were escorted into the Dynamic Memory Lab testing space where they were briefed on the experiment, and gave written informed consent. Participants then completed an MRI screening form and demographics questionnaires. Upon successful screening for the experiment, participants were escorted to the Imaging Research Center, and were prepped to undergo scanning. In addition to the primary experimenter, another member of the research team was always present during data collection. Experimenters were not blinded to any aspects of the experimental design. Scanning took place in a Siemens 3T Skyra with a 32-channel head coil. Participants were fitted with MRI-compatible earbuds with replaceable foam inserts (MRIaudio), and were provided with additional foam padding inside the head coil for hearing protection and to mitigate head motion. Participants were additionally given bodily padding, blankets, and corrective lenses as needed. An MRI-compatible microphone (Optoacoustics FOMRI-III) with bidirectional noise-cancelling was affixed to the head coil, and the receiver (covered by a disposable sanitary pop screen) was positioned over the participant's mouth. Participants were given a description of strategies to remain still while speaking during functional image acquisition. During MRI data acquisition, an eye tracker was operational to monitor participants' wakefulness and head motion during spoken recall, but no eye tracking data were recorded. After testing the earbuds and microphone, high-resolution T1-weighted structural images were acquired using a magnetization prepared rapid acquisition gradient echo (MPRAGE) pulse sequence (FOV = 256mm, image matrix = 256 x 256, sagittal slices = 208, thickness = 1mm). Participants then completed a functional imaging run in which they were shown still images of the central people and contexts subsequently shown in the videos (these data are not analyzed or discussed here). Participants then completed 3 runs in which all 8 event videos were shown, in random order across runs and across participants. Video stimuli (title screen + event) were 40 seconds, with a 10 second inter-stimulus interval displaying a fixation cross. Instructions were to remain still and closely attend to the videos, as memory for the videos would be tested later in the experiment. After the 3 encoding runs were completed, participants completed spoken recall of each event. Event titles were displayed in random order, in white text against a black background for 40 seconds, with a 10 second inter-stimulus interval displaying a fixation cross. Participants were instructed to begin recalling the named event, in as much detail as possible and in order to the extent possible, and to stop recall either when finished or when the event title transitioned to the fixation cross. Functional images were acquired using a multi-band gradient echo planar imaging (EPI) sequence (TR = 1220ms, TE = 24ms, FOV = 192mm, image matrix = 64 x 64, flip angle = 67, multi-band factor = 2, axial slices = 38, voxel size = 3mm isotropic, P >> A phase encoding, AC-PC alignment). A single 4 TR functional scan of reverse phase encoding polarity (A >> P) was acquired for unwarping.
Timing	Data were collected between approximately June 2018 through February 2019 on a rolling basis.
Data exclusions	We initially collected 24 participants, and excluded 1 due to falling asleep during MRI scanning, 2 due to excessive motion in the MRI scanner, and 1 due to equipment malfunction during data collection. The final sample of 20 participants had a mean age of 23.4 years (SD = 3.1 years), consisting of 13 self-identified females and 7 self-identified males.
Non-participation	No participants declined participation.
Randomization	As this was a within-subjects manipulation with random assignment of conditions per subject, no between-subjects randomization was necessary.

Reporting for specific materials, systems and methods

We require information from authors about some types of materials, experimental systems and methods used in many studies. Here, indicate whether each material, system or method listed is relevant to your study. If you are not sure if a list item applies to your research, read the appropriate section before selecting a response.

Materials & experimental systems

n/a	Involved in the study
☒	☐ Antibodies
☒	☐ Eukaryotic cell lines
☒	☐ Palaeontology and archaeology
☒	☐ Animals and other organisms
☐	☒ Human research participants
☒	☐ Clinical data
☒	☐ Dual use research of concern

Methods

n/a	Involved in the study
☒	☐ ChIP-seq
☒	☐ Flow cytometry
☐	☒ MRI-based neuroimaging

Human research participants

Policy information about studies involving human research participants

Population characteristics	See above.
Recruitment	Participants were recruited from the University of California, Davis and the Davis, California community via flyer and email advertisement. Participants were paid $25 per hour, and gave written informed consent in accordance with the University of California, Davis Institutional Review Board under an approved protocol. Given that participants were recruited on a volunteer basis from UC Davis and the surrounding community, there is a possibility that a sampling bias for college-aged, middle-class young adults is present that may not generalize to all populations.
Ethics oversight	The University of California, Davis Institutional Review Board approved of all procedures in the study.

Note that full information on the approval of the study protocol must also be provided in the manuscript.

Magnetic resonance imaging

Experimental design

Design type	Event-related task fMRI.
Design specifications	Encoding of video material: 3 runs each depicting 8 video clips, 40 seconds in duration, in randomized order. Recall of video material: one run of spoken recall of each video clip, 40 seconds in duration. For both encoding and recall, an inter-trial-interval of 10 seconds was inserted between events.
Behavioral performance measures	In-scanner recall was scored using an adapted version of the Autobiographical Memory Interview65,66(see Cohn-Sheehy et al. for a highly similar adapted approach56). Three scorers transcribed the audio recording for each event into text, and segmented the document into meaningful detail units (Z.M.R. and two research assistants). Detail units refers to the smallest possible meaningful unit, and labels were assigned to those details on the basis of their content. These details were then classified as "verifiable" if they referred to a factually accurate piece of information pertaining to the events depicted in the videos, and were not preceded by statements of uncertainty (e.g., "maybe"). Redundant information was not counted. Once scoring was completed, interrater reliability was assessed, and was fairly high across the three raters overall (Pearson r = 0.86), and in terms of details scored as being verifiable (Pearson r = 0.84). Verifiable details were compared across characters and across contexts using one-way ANOVAs, and were correlated with pattern similarity and recall-driven reinstatement using Pearson correlations. Out-of-scanner recognition test results for each participant considered distinction of true statements from false statements about the viewed events. To quantify this, we calculated a d' score for each participant: z(Hit rate) – z(False Alarm rate). Similar to in-scanner recall performance above, recognition memory scores were compared across characters and across contexts using one-way ANOVAs, and standard Pearson correlations were used to assess relationships with neural data across events. The ultimate goal of these analyses was to ensure that recognition and recall of event information were significantly nonzero, and unbiased across the events.

Acquisition

Imaging type(s)	Functional.
Field strength	3T.
Sequence & imaging parameters	Functional images were acquired using a multi-band gradient echo planar imaging (EPI) sequence (TR = 1220ms, TE = 24ms, FOV = 192mm, image matrix = 64 x 64, flip angle = 67, multi-band factor = 2, axial slices = 38, voxel size = 3mm isotropic, P >> A phase encoding, AC-PC alignment). A single 4 TR functional scan of reverse phase encoding polarity (A >> P) was acquired for unwarping.
Area of acquisition	Whole-brain.

Diffusion MRI ☐ Used ☒ Not used

Preprocessing

Preprocessing software	Preprocessing: FMRIPREP (RRID:SCR_016216). See Methods in manuscript for full list of sub-routines.
Normalization	antsRegistration tool of ANTs v2.1.075 (RRID:SCR_004757).
Normalization template	ICBM 152 Nonlinear Asymmetrical template version 2009c74 (RRID:SCR_008796).
Noise and artifact removal	Physiological noise regressors: first six principal components via CompCor were modeled as nuisance regressors. Motion: BOLD-to-T1w transformation and T1w-to-template (MNI) warp were concatenated and applied in a single step using antsApplyTransforms (ANTs v2.1.0) using Lanczos interpolation, and 6 resultant refitting parameters (i.e., motion parameters) were modeled as nuisance regressors.
Volume censoring	No censoring routines were used.

Statistical modeling & inference

Model type and settings	All representational similarity analyses (RSA) were run on unsmoothed native-space functional images after the preprocessing steps described above. Each of the 3 encoding runs and the recall run of the raw data were entered into a general linear model (GLM) in AFNI using the 3dDeconvolve function. Nuisance regressors for linear scanner drift (first order polynomial), head motion (6 directions plus their 6 derivatives), and the first two principal components of a combined white matter and CSF mask (via aCompCor above). Given the relatively long 40 second duration of the 'trials' being modeled and the 10 second gap between the videos, a least-squares all (LSA) approach was used, such that each run was modeled to produce beta estimate images for either encoding or recall of each of the 8 events. Though a number of prior studies examining naturalistic encoding and recall have not used GLMs to model data, and have instead used a time-shifted averaging approach, we chose to model the present data as this allowed us to more directly mitigate influences of signal drift, motion, and global nuisance signal in evaluating representational patterns. As events, including title screens, were 40 seconds in duration and the TR for our EPI sequence was 1.22 seconds, there were approximately 33 TRs covering each event. Encoding-encoding analyses simply computed event-by-event pattern similarity across runs, such that each event pattern similarity comparison was the average of three across-run comparisons (i.e., run1+run2, run1+run3, run2+run3). Correlation coefficients were then z-scored prior to model matrix comparisons or event-type analyses. Encoding-recall comparisons were followed the same principle as encoding-encoding comparisons, except the 33-TR events modeled at encoding were compared to variable TR events during recall. Specifically, the length of the recall epoch was defined as the duration of spoken recall for each participant (e.g., if a participant spoke for 18 TRs for a given event, the regressor for that event covered those 18 TRs). Recall betas were then compared to encoding betas across all 3 encoding runs.
Effect(s) tested	Primary analyses compared event-by-event correlation matrices to hypothesized model matrices using point-biserial correlations. These correlation coefficients (i.e., model fits) were t-tested against a null fit of zero, and were further compared to one another within-region using one-way ANOVAs and post hoc Tukey's HSD tests.

Specify type of analysis: ☐ Whole brain ☒ ROI-based ☐ Both

Anatomical location(s): Angular gyrus (ANG), posterior-medial cortex (PMC), parahippocamapal cortex (PHC), perirhinal cortex (PRC), temporal poles (TP), hippocampus (HPC), and medial prefrontal cortex (mPFC).

Statistic type for inference (See Eklund et al. 2016)	Cluster-wise analyses were not conducted.
Correction	Correction for model fits within-region used Tukey's HSD following a significant one-way ANOVA. As we did not test across-region hypotheses or conduct voxelwise analyses, no further corrections were applied.

Models & analysis

n/a	Involved in the study
☒	☐ Functional and/or effective connectivity
☒	☐ Graph analysis
☒	☐ Multivariate modeling or predictive analysis
